# Pathology of Ketoacidosis in Emergency of Diabetic Ketoacidosis and Alcoholic Ketoacidosis: A Retrospective Study

**DOI:** 10.1155/2024/8889415

**Published:** 2024-01-08

**Authors:** Katsumasa Koyama, Takatoshi Anno, Yukiko Kimura, Fumiko Kawasaki, Kohei Kaku, Koichi Tomoda, Hideaki Kaneto

**Affiliations:** ^1^Department of General Internal Medicine 1, Kawasaki Medical School, Okayama 700-8505, Japan; ^2^Department of Diabetes, Metabolism and Endocrinology, Kawasaki Medical School, Kurashiki 701-0192, Japan

## Abstract

This study is aimed at examining which factors are useful for the diagnosis and distinction of ketoacidosis. We recruited 21 diabetic ketoacidosis (DKA) and alcoholic ketoacidosis (AKA) patients hospitalized in Kawasaki Medical School General Medical Center from April 2015 to March 2021. Almost all patients in this study were brought to the emergency room in a coma and hospitalized. All patients underwent blood gas aspiration and laboratory tests. We evaluated the difference in diagnosis markers in emergencies between DKA and alcoholic ketoacidosis AKA. Compared to AKA patients, DKA patients had statistically higher values of serum acetoacetic acid and lower values of serum lactate, arterial blood pH, and base excess. In contrast, total ketone bodies, *β*-hydroxybutyric acid, and *β*-hydroxybutyric acid/acetoacetic acid ratio in serum did not differ between the two patient groups. It was shown that evaluation of each pathology such as low body weight, diabetes, liver dysfunction, and dehydration was important. It is important to perform differential diagnosis for taking medical histories such as insulin deficiency, alcohol abuse, or starvation as the etiology in Japanese subjects with DKA or AKA. Moreover, it is important to precisely comprehend the pathology of dehydration and alcoholic metabolism which would lead to appropriate treatment for DKA and AKA.

## 1. Introduction

Total ketone bodies include 3 molecules, acetoacetate (AcAc), *β*-hydroxybutyrate (*β*-OHB), and acetone. Under starvation conditions, especially glucose deficiency conditions, ketone bodies provide the brain with an important alternative metabolic fuel source. *β*-OHB play a key role in sparing glucose utilization and reducing proteolysis [1]. The accumulation of ketone bodies can be brought about in pathological states, which sometimes leads to metabolic acidosis with a high anion gap. The disorders related to ketone body accumulation are mainly classified by 3 distinct mechanisms: diabetic ketoacidosis (DKA), alcoholic ketoacidosis (AKA), and starvation ketoacidosis (SKA) [2]. Although these pathologies are similar as metabolic acidosis and increased total ketone body concentrations, there is a difference in the findings for diagnosis such as a clear history of diabetes mellitus, alcohol abuse, or starvation [3, 4].

DKA is one of the most serious acute metabolic complications of diabetes mellitus (DM), and the main mechanism of DKA is a lack of insulin in the body [5]. The criteria for diagnosing DKA include plasma glucose, >250 mg/dL; pH, <7.30; serum bicarbonate (HCO3-) level, <18 mEq/L; and the presence of ketonuria and/or ketonemia, an anion gap, >10 mEq/L [6]. However, since metabolic markers associated with DKA can be affected by various degrees of respiratory compensation or other metabolisms, they are relatively nonspecific for DKA [7].

AKA was first reported by Dillon et al. in 1940 [8]. The criteria for diagnosing AKA are as follows: AKA is a relatively common syndrome in chronic alcohol abusers and binge drinkers, and AKA is accompanied by metabolic acidosis and anion gap opening together with increased ketone body production triggered by chronic excessive alcohol consumption, withdrawal, or poor food intake, and dehydration [9, 10]. The clinical features of AKA are very similar to those of DKA, except for a history of chronic alcohol abuse and lack of specific clinical presentation [11, 12].

Most simple diagnosis of DKA or AKA is performed by taking the medical history of diabetes mellitus or alcohol abuse. However, in emergencies, patients with either DKA or AKA often suffer from disturbance of consciousness, and it is difficult to communicate with them. Additionally, DM patients who abuse alcohol can also develop AKA. Although there is no report showing a direct association between DM and AKA, there are a few reports that the two conditions can be coexistent [11, 12]. Although many cases of AKA are under poor conditions, individuals with AKA do not usually have an actual loss of consciousness despite the severity of acidosis and marked ketonemia [13]. When altered mental status and loss of consciousness are brought about, they are typically attributable to other underlying factors such as hypoglycemia or severe infection [12].

This study is aimed at examining which factors are useful for the diagnosis and distinction of simple DKA and simple AKA as possible in patients with ketoacidosis in emergencies. In this report, we present the characteristics of DKA and AKA to illustrate the importance of using a systematic approach to reach the final diagnosis.

## 2. Materials and Methods

### 2.1. Study Population

**Figure 1:**
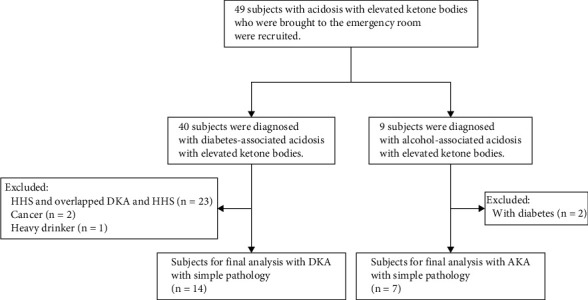
Flowchart of this study subjects. Abbreviations: DKA: diabetic ketoacidosis; AKA: alcoholic ketoacidosis; HHS: hyperosmolar hyperglycemic status.

The patients with ketoacidosis who were hospitalized in Kawasaki Medical School General Medical Center from April 2015 to March 2021 were included in this study. We recruited 49 subjects with ketoacidosis who were brought to the emergency room. First, we excluded patients with hyperosmolar hyperglycemic status (HHS) and overlapped DKA and HHS, because each pathology differed in ketoacidosis (*n* = 23). We excluded patients with cancer and/or using steroid drugs for the treatment of some diseases (*n* = 2). Almost all patients in this study were brought to the emergency room with coma and hospitalized. With the hope of providing a more complete approach pertaining to the biochemical analyses, we excluded AKA patients complicated with diabetes (*n* = 2) and DKA patient are heavy drinkers (*n* = 1).  Figure 1 shows the flowchart of selection and exclusion in this study subjects. This study protocol was approved by the Research Ethics Committee (REC) of Kawasaki Medical School and Hospital (protocol code 5770-00). Since this study was retrospective, instead of obtaining informed consent from each patient, we provided public information about this study via the hospital homepage.

### 2.2. Materials and Methods

Total ketone bodies, AcAc and *β*-OHB, were measured with an enzyme cycling method (FUJIFILM Wako Pure Chemical Co., Japan; JCA-ZS050, JEOL Ltd., Japan). pH, base excess (BE), and lactate were measured with a blood gas analysis (ABL-800 FLEX, Radiometer Medical ApS., Denmark). Chemistry examination (ChE, total protein, albumin, total cholesterol, triglyceride, *γ*-glutamyl transpeptidase (*γ*-GTP), aspartate aminotransferase (AST), alanine aminotransferase (ALT), creatinine, urea nitrogen, sodium, potassium, chloride, and C-reactive protein (CRP)) was performed with an enzyme method and ion-selective electrode method (Shino-Test Co., Japan; Sekisui Medical Co., Ltd., Japan; KAINOS Laboratories, Inc., Japan; and JCA-ZS050, JEOL Ltd., Japan). Plasma glucose, HbA1c, and blood osmotic pressure were measured with an amperometry method, high-performance liquid chromatography (HPLC) method, and cryoscopic method (ADAMS Glucose GA-1172, ADAMS A1c HA-8182, and OSMO STATION OM-6060, ARKRAY, Inc., Japan).

### 2.3. Statistical Analysis

All analyses were performed by using JMP version 9 (SAS Institute Inc.). The Wilcoxon rank sum test was performed to examine the various clinical parameters in comparison of DKA and AKA.

## 3. Results

### 3.1. Characteristics of the Study Patients

The clinical characteristics of the patients in this study are shown in Table 1. The clinical parameters in DKA and AKA patients were as follows: total ketone bodies (reference range, 0.0–130.0 *μ*mol/L), 11029.2 ± 1957.4 *μ*mol/L vs. 5737.8 ± 1964.0 *μ*mol/L; AcAc (reference range, 0.0–55.0 *μ*mol/L), 2716.4 ± 493.2 *μ*mol/L vs. 1073.1 ± 258.3 *μ*mol/L (*p* < 0.05); *β*-OHB (reference range, 0.0–85.0 *μ*mol/L), 8310.7 ± 1542.5 *μ*mol/L vs. 4837.8 ± 1762.1 *μ*mol/L; *β*-OHB/AcAc, 3.11 ± 0.43 vs. 4.31 ± 0.84; pH (reference range, 7.360–7.460), 7.00 ± 0.04 vs. 7.20 ± 0.03 (*p* < 0.05); and BE (reference range, -2.5–2.5 mEq/L), −26.6 ± 2.4 mEq/L vs. −13.5 ± 1.6 mEq/L (*p* < 0.05). These data suggest the presence of marked elevation of ketone bodies and acidosis in both groups. Lactate levels were markedly higher in AKA compared to DKA: lactate (reference range, 0.63–2.44 mEq/L), 2.61 ± 0.42 mEq/L vs. 12.01 ± 2.80 mEq/L (*p* < 0.0005) in DKA and AKA, respectively.

Also, we divided male and female data to better understand sex-related effects on the development of ketoacidosis, which can also be included in the discussion. The clinical characteristics of the patients in this study are shown in Table 2. The clinical parameters with sex-related effects in DKA patients were as follows (male vs. female): total ketone bodies, 10311.5 ± 2602.4 *μ*mol/L vs. 11348.2 ± 2670.5 *μ*mol/L; AcAc, 2509.4 ± 495.2 *μ*mol/L vs. 2808.5 ± 694.7 *μ*mol/L; *β*-OHB, 7795.4 ± 2130.1 *μ*mol/L vs. 8539.7 ± 2092.3 *μ*mol/L; *β*-OHB/AcAc, 2.96 ± 0.32 vs. 3.18 ± 0.62; pH, 7.11 ± 0.06 vs. 6.94 ± 0.05 (*p* < 0.05); base excess (BE), −21.9 ± 3.7 mEq/L vs. −29.2 ± 2.8 mEq/L (*p* < 0.05); and lactate, 2.61 ± 0.42 mEq/L vs. 2.70 ± 0.57 mEq/L in male and female, respectively. Similarly, the clinical parameters with sex-related effects in AKA patients were as follows (male vs. female): total ketone bodies, 2842.1 ± 537.8 *μ*mol/L vs. 8633.5 ± 3257.6 *μ*mol/L; AcAc, 421.5 ± 249.6 *μ*mol/L vs. 1378.5 ± 205.7 *μ*mol/L (*p* < 0.05); *β*-OHB, 2420.6 ± 579.5 *μ*mol/L vs. 7254.9 ± 3057.3 *μ*mol/L; *β*-OHB/AcAc, 3.44 ± 0.74 vs. 4.89 ± 1.32; pH, 7.23 ± 0.05 vs. 7.18 ± 0.03; base excess (BE), −11.0 ± 2.3 mEq/L vs. −15.3 ± 1.8 mEq/L; and lactate, 13.95 ± 3.91 mEq/L vs. 8.14 ± 1.86 mEq/L in male and female, respectively.

After that, we compared the clinical parameters in DKA and AKA patients with divided male and female data. The clinical parameters of male in DKA and AKA patients were as follows: total ketone bodies, 10311.5 ± 2602.4 *μ*mol/L vs. 2842.1 ± 537.8 *μ*mol/L (*p* < 0.05); AcAc, 2509.4 ± 495.2 *μ*mol/L vs. 421.5 ± 249.6 *μ*mol/L (*p* < 0.05); *β*-OHB, 7795.4 ± 2130.1 *μ*mol/L vs. 2420.6 ± 579.5 *μ*mol/L (*p* < 0.05); *β*-OHB/AcAc, 2.96 ± 0.32 vs. 3.44 ± 0.74; pH, 7.11 ± 0.06 vs. 7.23 ± 0.05; and base excess (BE), −21.9 ± 3.7 mEq/L vs. −11.0 ± 2.3 mEq/L. These data suggest the presence of marked elevation of ketone bodies and acidosis in both groups in male and that ketone body levels were markedly higher in DKA compared to AKA. Lactate levels were markedly higher in AKA compared to DKA: lactate, 2.61 ± 0.42 mEq/L vs. 13.95 ± 3.91 mEq/L (*p* < 0.05) in DKA and AKA, respectively. In addition, the clinical parameters of female in DKA and AKA patients were as follows: total ketone bodies, 11348.2 ± 2670.5 *μ*mol/L vs. 8633.5 ± 3257.6 *μ*mol/L; AcAc, 2808.5 ± 694.7 *μ*mol/L vs. 1378.5 ± 205.7 *μ*mol/L; *β*-OHB, 8539.7 ± 2092.3 *μ*mol/L vs. 7254.9 ± 3057.3 *μ*mol/L; *β*-OHB/AcAc, 3.18 ± 0.62 vs. 4.89 ± 1.32; pH, 6.94 ± 0.05 vs. 7.18 ± 0.03 (*p* < 0.005); and base excess (BE), −29.2 ± 2.8 mEq/L vs. −15.3 ± 1.8 mEq/L. These data suggest the presence of marked elevation of ketone bodies and acidosis in both groups in female and that acidosis levels were markedly severe in DKA compared to AKA. Lactate levels were markedly higher in AKA compared to DKA: lactate, 2.70 ± 0.57 mEq/L vs. 8.14 ± 1.86 mEq/L (*p* < 0.005) in DKA and AKA, respectively.

### 3.2. Low Body Weight in Patients with Alcoholic Ketoacidosis

**Figure 2:**
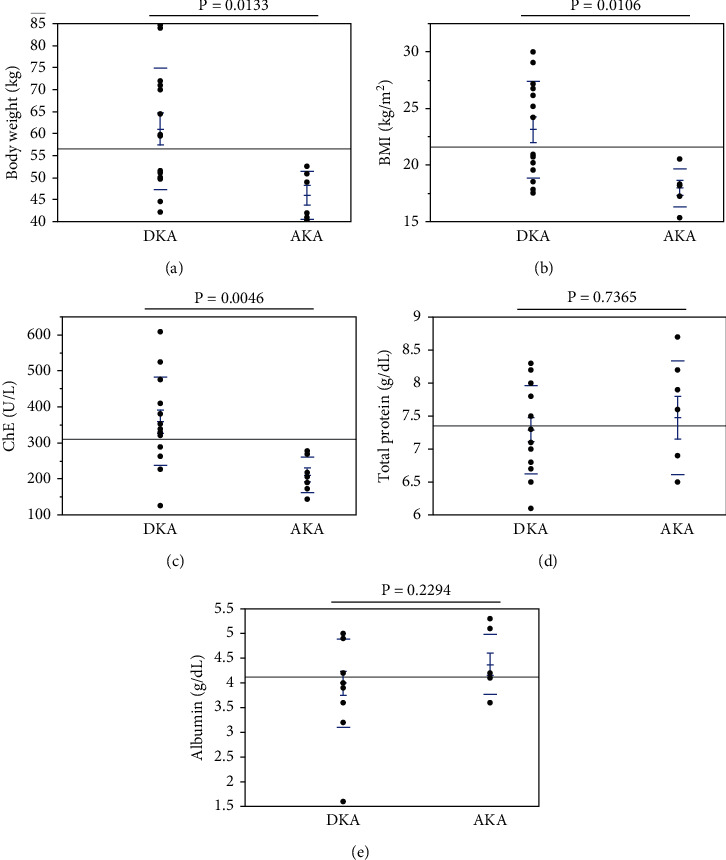
Body weight in patients with DKA and AKA: (a) body weight; (b) BMI; (c) ChE; (d) total protein; (e) albumin. There was a very close association between AKA and various factors related to low body weight and hypoalbuminemia. DKA patients had statistically higher values of body weight, BMI, and ChE than AKA patients, while total protein and albumin did not differ between the two patient groups. Abbreviations: DKA: diabetic ketoacidosis; AKA: alcoholic ketoacidosis; BMI: body mass index; ChE: cholinesterase.

There was a very close association between AKA and various factors such as low body weight (Figure 2). The parameters in DKA and AKA patients were as follows: body weight, 61.1 ± 3.7 kg vs. 46.0 ± 2.2 kg (*p* < 0.05) (Figure 2(a)); body mass index (BMI), 23.1 ± 1.1 kg/m^2^ vs. 18.0 ± 0.7 kg/m^2^ (*p* < 0.05) (Figure 2(b)); and cholinesterase (ChE) (reference range, 240–486 U/L), 359.4 ± 32.8 U/L vs. 211.1 ± 18.5 U/L (*p* < 0.005) (Figure 2(c)). There was no difference in total protein (Figure 2(d)) and albumin levels (Figure 2(e)) between DKA and AKA. These data indicate that in AKA patients, carbohydrate and protein stores were markedly depleted due to chronic alcohol misusers.

### 3.3. Metabolic Markers in Patients with Alcoholic Ketoacidosis and Diabetic Ketoacidosis

**Figure 3:**
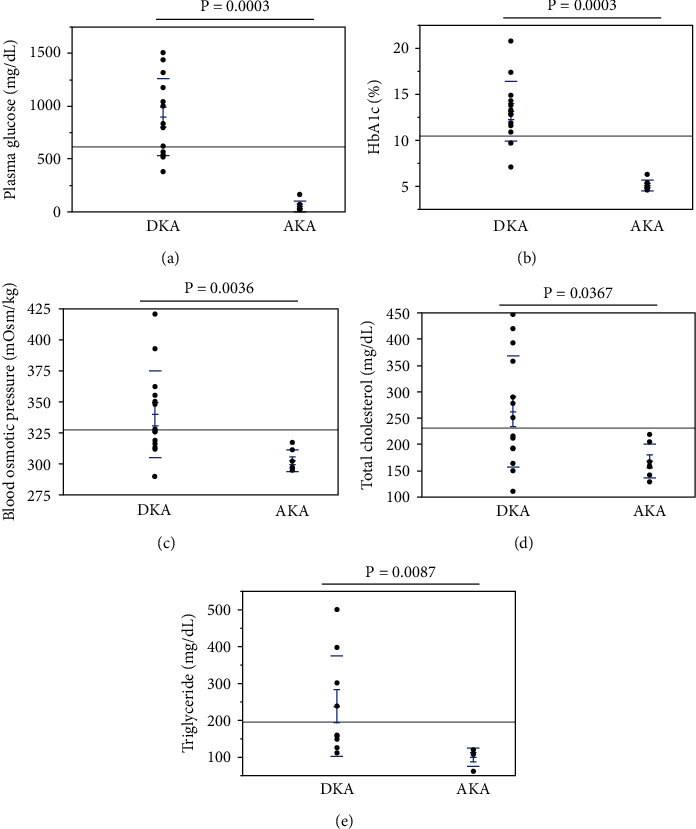
Metabolic markers in patients with DKA and AKA: (a) plasma glucose; (b) HbA1c; (c) blood osmotic pressure; (d) total cholesterol; (e) triglyceride. There was a very close association between AKA and various factors related to metabolic markers. DKA patients had statistically higher values of plasma glucose levels, HbA1c, serum osmolality, total cholesterol, and triglyceride than AKA patients, while total protein, albumin, and beta-hydroxybutyric acid/acetoacetic acid ratio in serum did not differ between the two patient groups. Abbreviations: DKA: diabetic ketoacidosis; AKA: alcoholic ketoacidosis; HbA1c: hemoglobin A1c.

DKA patients had severe hyperglycemia and poorly controlled DM. Almost all AKA patients were under hypoglycemic conditions and were brought to the emergency room with coma (Figure 3). The parameters in DKA and AKA patients were as follows: plasma glucose levels, 896.9 ± 96.7 mg/dL vs. 51.0 ± 20.3 mg/dL (*p* < 0.0005) (Figure 3(a)); hemoglobin A1c (HbA1c) (reference range, 4.9–6.0%), 13.2 ± 0.9% vs. 5.1 ± 0.2% (*p* < 0.0005) (Figure 3(b)); and serum osmolality (reference range, 275–290 mOsm/kg), 340.0 ± 9.3 mOsm/kg vs. 302.3 ± 3.3 mOsm/kg (*p* < 0.005) (Figure 3(c)). Lipid markers were also lower in AKA compared to DKA (Figure 3): total cholesterol (reference range, 142–248 mg/dL), 262.5 ± 28.3 mg/dL vs. 168.4 ± 12.3 mg/dL (*p* < 0.05) (Figure 3(d)), and triglyceride (reference range, 30–149 mg/dL), 238.3 ± 45.3 mg/dL vs. 99.8 ± 12.9 mg/dL (*p* < 0.05) (Figure 3(e)). These data indicate that glycemic control was quite poor in DKA patients. On the other hand, hypoglycemia and low levels of lipid-associated data in AKA patients may reflect low body weight.

### 3.4. Liver Dysfunction due to Heavy Drinking in Patients with Alcoholic Ketoacidosis

**Figure 4:**
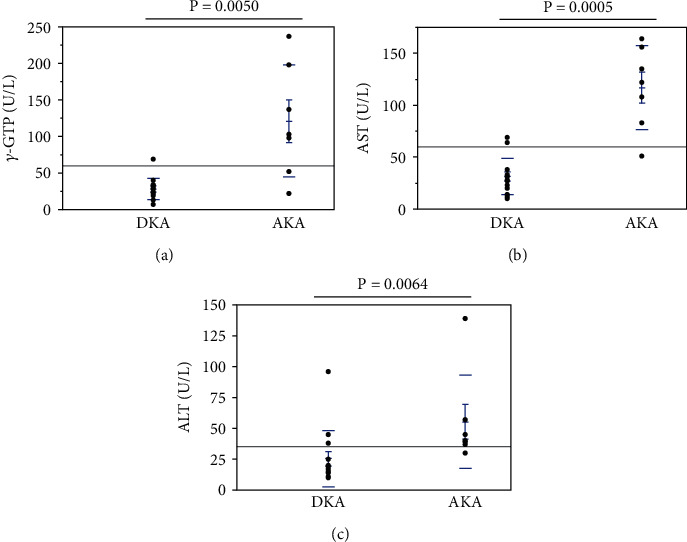
Hepatic markers in patients with DKA and AKA: (a) *γ*-GTP; (b) AST; (c) ALT. Since patients with AKA were heavy drinkers, they had liver dysfunction. DKA patients had statistically lower values of *γ*-GTP, AST, and ALT than AKA patients. Abbreviations: DKA: diabetic ketoacidosis; AKA: alcoholic ketoacidosis; *γ*-GTP: *γ*-glutamyl transpeptidase; AST: aspartate aminotransferase; ALT: alanine aminotransferase.

AKA patients had liver dysfunction due to heavy drinking (Figure 4). The parameters related to liver function in DKA and AKA patients were as follows: *γ*-glutamyl transpeptidase (*γ*-GTP) (reference range, 9–32 U/L), 27.9 ± 3.9 U/L vs. 121.0 ± 28.9 U/L (*p* < 0.005) (Figure 4(a)); aspartate aminotransferase (AST) (reference range, 13–30 U/L), 31.3 ± 4.6 U/L vs. 117.0 ± 15.2 U/L (*p* < 0.0005) (Figure 4(b)); and alanine aminotransferase (ALT) (reference range, 7–23 U/L), 25.4 ± 6.1 U/L vs. 55.3 ± 14.3 U/L (*p* < 0.005) (Figure 4(c)). Liver dysfunction in AKA patients may reflect alcoholic liver damage.

### 3.5. Other Biochemical Markers in Patients with Alcoholic Ketoacidosis

**Figure 5:**
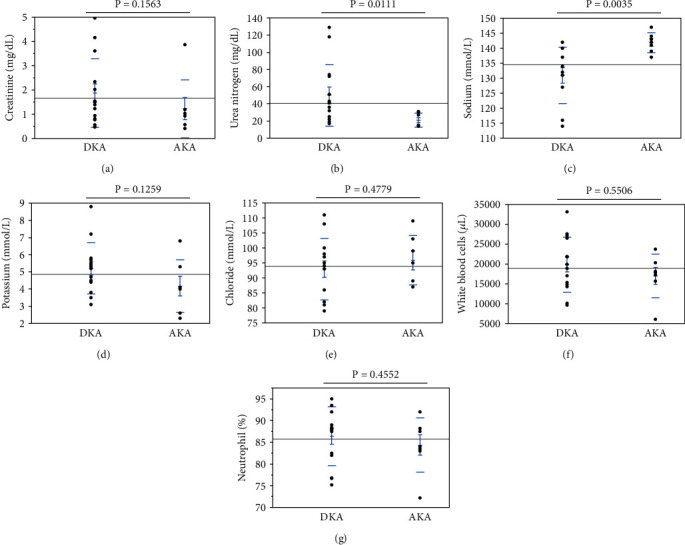
Renal markers, mineral balance markers, and bloody cell counts in patients with DKA and AKA: (a) creatinine; (b) urea nitrogen; (c) sodium; (d) potassium; (e) chloride; (f) white blood cells; (g) neutrophils. DKA patients had statistically higher values of urea nitrogen than AKA patients, while creatinine levels did not differ between the two patient groups. Regarding electrolytes, there was a difference only in sodium level. DKA patients had statistically higher serum sodium levels than AKA patients, while potassium and chloride in serum did not differ between the two patient groups. Moreover, white blood cells and neutrophils did not differ between the two patient groups. Abbreviations: DKA: diabetic ketoacidosis; AKA: alcoholic ketoacidosis.

It seems that both DKA and AKA cause dehydration, which is associated with diuretic effects by hyperglycemia or alcohol, although this is no conclusive evidence about this point. Creatinine levels were not different between DKA and AKA. Urea nitrogen (BUN) levels were lower in AKA compared to DKA (Figure 5). The parameters related to kidney function in DKA and AKA patients were as follows: creatinine (reference range, 0.46–0.79 mg/dL), 1.88 ± 0.38 mg/dL vs. 1.23 ± 0.36 mg/dL (Figure 5(a)), and BUN (reference range, 8–20 mg/dL), 50.1 ± 9.6 mg/dL vs. 20.9 ± 3.0 mg/dL (*p* < 0.05) (Figure 5(b)). Electrolytes in DKA and AKA patients were as follows: sodium (reference range, 138–145 mmol/L), 130.9 ± 2.5 mmol/L vs. 141.9 ± 1.2 mmol/L (*p* < 0.005) (Figure 5(c)); potassium (reference range, 3.6–4.8 mmol/L), 5.21 ± 0.39 mmol/L vs. 4.17 ± 0.58 mmol/L (Figure 5(d)); and chloride (reference range, 101–108 mmol/L), 92.9 ± 2.7 mmol/L vs. 95.9 ± 3.1 mmol/L (Figure 5(e)). White blood cells and neutrophil levels were markedly elevated compared with normal range in both groups, but there was no significant difference between DKA and AKA (Figures 5(f) and 5(g)). Hemoglobin, hematocrit, and C-reactive protein (CRP) levels were not different between DKA and AKA. In addition, amylase and pancreatic-amylase levels were not different between DKA and AKA. It seems that the changes in BUN and sodium indicate that dehydration and electrolyte abnormalities are associated, at least in part, with the pathophysiology, although each pathophysiology differs in DKA and AKA.

## 4. Discussion

The differential diagnosis of conditions resulting in high anion gap is difficult and complicated, especially when the conditions are accompanied by various metabolic disorders including ketoacidosis (DKA, AKA, and SKA), renal failure, lactic acidosis, and various ingestions (methanol, ethylene glycol, salicylates, etc.). When dealing with ketoacidosis, taking a medical history is important to perform differential diagnoses such as insulin deficiency, alcohol abuse, or starvation as the etiology. However, it is also undeniable that some patients have the effects of both, such as DKA who are heavy alcohol drinkers and AKA undergoing diabetes treatment.

DKA results in a loss of consciousness, and AKA also results in impaired consciousness. AKA sometimes leads to the reason for the investigation, admission, and sudden unexplained deaths of alcohol-dependent patients, which has been reported worldwide, especially in Europe and the USA [12, 14]. On the other hand, AKA is very rarely diagnosed as checked acidosis caused by alcohol in Japan. This may be due to the difference in the number of emergency department visits by alcoholics between Japan and the US, as well as the fact that the concept of AKA is not widely accepted among clinicians in Japan [15]. In this study, 5 of the 7 AKA patients were transported to the emergency room because of hypoglycemia. It seems that many typical AKA patients are complicated with hypoglycemia, which leads to disturbance of consciousness and needs transportation to the emergency room in Japan. In addition, such cases of hypoglycemia were complicated by low body weight due to heavy drinking. Thus, the disturbance of consciousness complicated with AKA is smoothly improved as hypoglycemia is improved. Therefore, when an AKA patient presents with hyperglycemia, the coexistence of another condition such as DKA must be considered. In this study, we focus specifically on a simple comparison of cases of each DKA and AKA, which was complicated with other pathology. It is known that we should pay attention to diagnosis especially when both conditions are involved, since the cause of impaired consciousness is different between hyperglycemia with dehydration and hypoglycemia and since the treatments of first touch are quite different.

**Figure 6:**
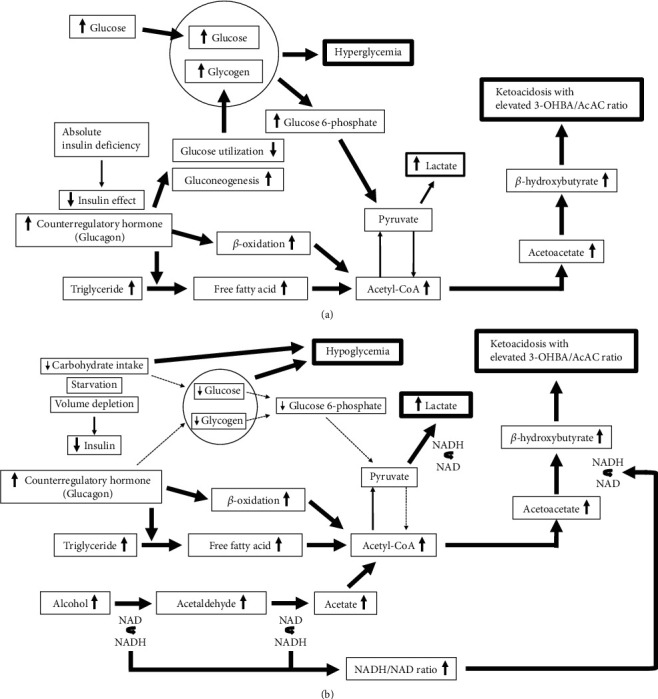
(a) Pathophysiology of diabetic ketoacidosis. DKA results in excessive amounts of glucose and glycogen due to absolute insulin deficiency, which finally leads to hyperglycemia. Hyperglycemia causes osmotic diuresis, leading to dehydration. In addition, in DKA, the enormous supply of fatty acyl CoA and deficiency in oxaloacetate overwhelm these normal biochemical pathways. Under the conditions, excessive amounts of acetyl-CoA derivatives are oxidized to form ketone bodies, and large quantities of *β*-OHB and AcAc are released into the blood stream. (b) Pathophysiology of alcoholic ketoacidosis. AKA is caused by various factors such as poor intake of carbohydrate and suppression of insulin with increased counterregulatory hormones. Low glycogen storage and suppression of gluconeogenesis lead to hypoglycemia. NAD is reduced to NADH during the metabolism of alcohol. Alcohol is metabolized to acetate, which consequently forms acetyl-CoA. AcAc tends to metabolize into *β*-OHB when a NADH/NAD ratio is high, which causes ketoacidosis with a high *β*-OHB/AcAc ratio.

One of important points is the ratio of *β*-OHB to AcAc (*β*-OHB/AcAc ratio) in ketoacidosis. There have been several previous reports on levels of *β*-OHB, levels of AcAc, and *β*-OHB/AcAc ratio in DKA and AKA. In general, AKA is often characterized by a high *β*-OHB/AcAc ratio with a high anion gap and elevated lactate. It has been reported that a *β*-OHB/AcAc ratio is significantly higher in AKA compared to DKA [16]. It was reported that this ratio averages about 5-7 in AKA and about 2-3 in DKA [12, 16]. In the present study, there was not a significant difference in *β*-OHB level and a *β*-OHB/AcAc ratio between DKA and AKA, although AcAc was significantly lower in AKA. As shown in the present study, since there was a significant difference in pH and BE between DKA and AKA, acidosis was severe in DKA (Table 1). These results are likely caused by a large amount of ketone bodies, although there was no significant difference between DKA and AKA. As described in the result section, there was a difference in the amount of ketone body levels, including AcAc and *β*-OHB, and the degree of acidosis. Additionally, it is known that the *β*-OHB/AcAc ratio changes markedly during acute DKA from a normal ratio of 1 : 1 to as high as 15 : 1 [17, 18]. Once we start insulin therapy, *β*-OHB levels usually decrease speedily before the AcAc level and this ratio returns to normal as *β*-OHB is metabolized to acetoacetate. Moreover, it has been reported that AKA patients have higher blood lactate concentrations and a higher lactate to pyruvate ratio compared to those with DKA [12, 19], although it was reported that lactate levels were elevated in DKA patients [20]. Additionally, lactate level was significantly elevated in AKA compared with DKA in the present study. The features of AKA such as a high *β*-OHB/AcAc ratio and elevated lactate may be able to explain the pathology of each DKA and AKA. As shown in Figure 6(a), the combination of insulin deficiency and increased counterregulatory hormones in DKA also leads to the release of free fatty acids into the circulation from adipose tissue and to hepatic fatty acid oxidation to ketone bodies. The alteration finally brings about severe ketonemia and severe metabolic acidosis [21]. On the other hand, as shown in Figure 6(b), it seems that the acidosis in AKA results from the accumulation in plasma lactate and ketone bodies including *β*-OHB and AcAc induced by increasing the nicotinamide-adenine dinucleotide (NADH)/nicotinamide-adenine dinucleotide (NAD) ratio [22]. The increased NADH/NAD ratio is thought to be pivotal in the development of ketoacidosis and lactic acidosis in AKA. The production of lactate and the degree of acidosis, which was associated with elevated lactate, may be caused by differences in pathophysiology, such as hyperglycemia in DKA and elevated NADH/NAD ratio in AKA, and which may be a useful factor in the differentiation. In the present study, since metabolic markers associated with ketoacidosis can be affected by various degree of respiratory compensation or other metabolisms, they are relatively nonspecific and lack specific clinical presentation. Therefore, it was very difficult to determine the pathophysiology based on only one point of examination in an emergency.

In this study, there was a significant difference in various clinical parameters between DKA and AKA. Although the sample size in this study was small, several differences were observed with divided male and female data. Not to mention, there was a significant difference in terms of physical stature in both DKA and AKA. These data suggest the presence of marked elevation of ketone bodies in males with DKA groups. On the other hand, these data suggest the presence of marked elevation of ketone bodies in females with AKA groups. However, although there were some gender differences compared with DKA and AKA, the results of the presence of significant differences in ketone bodies, acidosis, and lactate levels have remained in comparison to DKA and AKA. The most obvious difference is low body weight in AKA compared with DKA in the present study. Even SKA can lead to a high anion gap metabolic acidosis. In addition, starvation increases levels of cortisol and growth hormone [22]. Chronic alcohol misusers have markedly depleted carbohydrate and protein stores. In addition, although many chronic alcohol misusers may still receive some caloric intake from ethanol, other sources of dietary intake may be chronically reduced, resulting in starvation and depleted hepatic glycogen stores [23]. Glycogenesis is suppressed due to the increased NADH/NAD ratio [24]. Extremely low body weight patients may be more likely to develop under AKA conditions. Hypoglycemia may be a more common cause of impaired consciousness in such patients. In the present study, we showed that there was a very close association between AKA and various factors such as low body weight. Liver dysfunction may be pathological as a result of chronic alcoholism [24]. This study also showed significant liver dysfunction in AKA. On the other hand, as a matter of course, DKA patients suffered from DM and their glycemic control was poor in many cases.

Dehydration may occur in both DKA and AKA. However, creatinine levels were not different between DKA and AKA, although BUN levels were lower in AKA compared to DKA. Although dehydration is thought to be a factor in the development of AKA [9], there were no obvious findings of dehydration in this study. However, under DKA conditions, osmotic diuresis due to hyperglycemia and dehydration can occur in most circumstances [6]. It is thought that dehydration under DKA conditions complicated with hyperglycemia led to elevated BUN in this study, although we excluded hyperglycemic hyperosmolar state (HHS) patients, who were mainly diagnosed under pathological conditions. Since it was reported that dehydration with hyperglycemia led to elevated BUN levels, in the present study, elevated BUN may also reflect a condition of dehydration. In addition, electrolyte abnormality in AKA patients has been also described, although it was not observed systematically [12, 19]. It was reported that concurrent disease processes, including extracellular fluid depletion, alcohol withdrawal, and severe liver disease, resulted in various mineral balance abnormalities which mixed acid-base disturbances in AKA patients [9]. In this study, however, we revealed normal sodium levels in AKA and significantly decreased sodium levels in DKA. In general, conditions with hyperglycemia increase plasma osmolality and mobilize water from within the cells, resulting in a decreased serum sodium level. Isolated dehydration causes hypernatremia, not hyponatremia. Hyperglycemic crises cause both volume depletion and dehydration through osmotic diuresis. The loss of water in osmotic dehydration is relatively greater than the loss of sodium and potassium. The hyponatremia in hyperglycemia is the result of cellular dehydration. Volume depletion in AKA can be associated with either hyponatremia or hypernatremia when the rates of water and monovalent cation losses differ. Finally, when the blood level of urea is examined, both the rate of production and the rate of excretion of urea should be addressed [25]. This result may be due to the fact that the typical AKA is underweight and undernourished and has an obvious reduction in blood glucose, lipids, etc., which gives rise to colloid serum osmolality.

There are several strengths in this study. First, this report compared simple DKA with simple AKA, eliminating other factors as much as possible. In metabolism-associated status, many factors are involved and affect each other, complicating the pathophysiology. Therefore, despite the importance of comparing such DKA and AKA in the absence of other effects, there have been few reports. Second, awareness of AKA among the Japanese is low, and this study is especially important for the initial response in emergency settings. Third, despite the unique circumstances of diabetes and alcohol, DKA and AKA finally have become similar conditions as ketoacidosis. It was shown that evaluation of each pathology such as serum albumin value, diabetes, liver dysfunction, and dehydration was finally important.

There are limitations in this study. First, the sample size in this study was small, and this study was performed in Japanese subjects. Therefore, we think that a relatively small sample size can limit the generalizability and reliability of the findings in this study. In addition, we think that the results of this study are not necessarily applicable to Caucasians. Second, due to the limitation of the small sample size, the parameters were limited, such as free fatty acids, amylase, and pancreatic-amylase levels, to comprehend the complicated DKA and AKA conditions. Third, the study design is retrospective, meaning it relies on historical data. This can introduce potential biases and may not provide as strong evidence as prospective studies.

As shown in this study, while metabolic conditions, such as DKA and AKA, are complicated in their pathogenesis, SGLT2 inhibitors, which are often used for type 2 DM, are also associated with DKA. When using SGLT2 inhibitors, insulin secretion is reduced due to increase of urinary glucose excretion and decrease of blood glucose levels. Also, the amount of glucose which can be utilized in the body is decreased, and thereby, production of ketone body production is increased. In DKA triggered by the usage of SGLT2 inhibitors, blood glucose levels may not be significantly elevated due to increase of urinary glucose excretion (“euglycemic DKA”) [26]. Although there were no SGLT2 inhibitor users in this study, the pathogenesis of metabolic acidosis may be more complicated and require more attention in diabetic patients using SGLT2 inhibitors. In addition, this study focuses on DKA and AKA, with limited discussion of other potential conditions that can lead to high anion gap metabolic acidosis.

## 5. Conclusion

It would be safe to conclude that when dealing with ketoacidosis, taking a medical history is important to perform differential diagnosis such as insulin deficiency, alcohol abuse, or starvation as the etiology, rather than the degree of acidosis and *β*-OHB/AcAc ratio in Japanese subjects with DKA and AKA. The data in this study clearly show that it is important to precisely comprehend the pathology of dehydration and alcoholic metabolism which would lead to appropriate treatment for DKA and AKA. On the other hand, since the data in this study acknowledge that the pathogenesis of metabolic conditions such as DKA and AKA is quite complex, a more detailed investigation would be necessary in the future.

## Figures and Tables

**Table 1:** Comparison of various values between DKA and AKA patients on admission.

Parameters	DKA patients (*n* = 14)	AKA patients (*n* = 7)	*p* value
Male/female	5/9	4/3	
T1DM/T2DM	7/7	NA	
Age (years)	49.5 ± 4.7	55.3 ± 6.5	0.5253
Height (cm)	162.0 ± 1.9	160.0 ± 2.4	0.5743
Body weight (kg)	61.1 ± 3.7	46.0 ± 2.2	0.0133^∗^
BMI (kg/m^2^)	23.1 ± 1.1	18.0 ± 0.7	0.0106^∗^
Total ketone body (*μ*mol/L)	11029.2 ± 1957.4	5737.8 ± 1964.0	0.1144
Acetoacetic acid (*μ*mol/L)	2716.4 ± 493.2	1073.1 ± 258.3	0.0179^∗^
*β*-Hydroxybutyric acid (*μ*mol/L)	8310.7 ± 1542.5	4837.8 ± 1762.1	0.1605
*β*-OHB/AcAc	3.11 ± 0.43	4.31 ± 0.84	0.1265
pH	7.00 ± 0.04	7.20 ± 0.03	0.0133^∗^
BE (mEq/L)	−26.6 ± 2.4	−13.5 ± 1.6	0.0160^∗^
Lactate (mEq/L)	2.61 ± 0.42	12.01 ± 2.80	0.0005^∗^

Comparison of various values among patients with DKA and AKA in this study. Data presented as mean ± standard deviation. ^∗^*p* < 0.05 with the Wilcoxon rank sum test compared to DKA or AKA. Abbreviations: DKA: diabetic ketoacidosis; AKA: alcoholic ketoacidosis; T1DM: type 1 diabetes mellitus; T2DM: type 2 diabetes mellitus; BMI: body mass index; *β*-OHB: *β*-hydroxybutyrate; AcAc: acetoacetate; BE: base excess.

**Table 2:** Comparison of various values between DKA and AKA patients with divided males (M) and females (F).

Parameters	DKA (M) (*n* = 5)	DKA (F) (*n* = 9)	AKA (M) (*n* = 4)	AKA (F) (*n* = 3)	DKA (M) vs. (F) *p* value	AKA (M) vs. (F) *p* value	Males DKA vs. AKA *p* value	Females DKA vs. AKA *p* value
T1DM/T2DM	1/4	6/3	NA	NA				
Age (years)	49.2 ± 4.7	49.7 ± 4.4	51.3 ± 9.7	58.0 ± 9.9	0.4821	0.3753	0.4007	0.1958
Height (cm)	167.6 ± 1.9	158.8 ± 2.1	163.6 ± 2.3	154.5 ± 2.3	0.0095^∗^	0.0209^∗^	0.1127	0.1494
Body weight (kg)	68.1 ± 7.5	57.2 ± 3.6	47.3 ± 3.7	44.6 ± 3.1	0.0814^∗^	0.3048	0.0459^∗^	0.0457^∗^
BMI (kg/m^2^)	24.1 ± 2.4	22.6 ± 1.2	17.3 ± 1.0	18.7 ± 1.0	0.2685	0.1864	0.0318^∗^	0.0588
Total ketone body (*μ*mol/L)	10311.5 ± 2602.4	11348.2 ± 2670.5	2842.1 ± 537.8	8633.5 ± 3257.6	0.4094	0.0772^∗^	0.0308^∗^	0.3018
AcAc (*μ*mol/L)	2509.4 ± 495.2	2808.5 ± 694.7	421.5 ± 249.6	1378.5 ± 205.7	0.3965	0.0208^∗^	0.0101^∗^	0.1392
*β*-OHB (*μ*mol/L)	7795.4 ± 2130.1	8539.7 ± 2092.3	2420.6 ± 579.5	7254.9 ± 3057.3	0.4174	0.0976	0.0452^∗^	0.3791
*β*-OHB/AcAc	2.96 ± 0.32	3.18 ± 0.62	3.44 ± 0.74	4.89 ± 1.32	0.4103	0.2389	0.2523	0.1092
pH	7.11 ± 0.06	6.94 ± 0.05	7.23 ± 0.05	7.18 ± 0.03	0.0238^∗^	0.2360	0.1244	0.0008^∗^
BE (mEq/L)	−21.9 ± 3.7	−29.2 ± 2.8	−11.0 ± 2.3	−15.3 ± 1.8	0.0722	0.1129	0.0723	0.0101^∗^
Lactate (mEq/L)	2.61 ± 0.42	2.70 ± 0.57	13.95 ± 3.91	8.14 ± 1.86	0.3977	0.1931	0.0068^∗^	0.0022^∗^

Comparison of various values among patients with DKA and AKA with divided males (M) and females (F) in this study. Data presented as mean ± standard deviation. ^∗^*p* < 0.05 with the Wilcoxon rank sum test compared to DKA or AKA. Abbreviations: M: males; F: females; DKA: diabetic ketoacidosis; AKA: alcoholic ketoacidosis; T1DM: type 1 diabetes mellitus; T2DM: type 2 diabetes mellitus; BMI: body mass index; *β*-OHB: *β*-hydroxybutyrate; AcAc: acetoacetate; BE: base excess.

## Data Availability

The data underlying this article cannot be shared publicly due to privacy concerns. The data could be available after appropriate ethical committee approval and should be handled in compliance with relevant data protection and privacy regulations.
